# Supplementary material for: Mechanistic synergy of hair growth promotion by the Avicennia marina extract and its active constituent (avicequinone C) in dermal papilla cells isolated from androgenic alopecia patients
Source: PLoS One. 2023 Apr 21;18(4):e0284853. doi: 10.1371/journal.pone.0284853 (PMC10121027; doi:10.1371/journal.pone.0284853)
Supplement: S1 File — (ZIP) [file pone.0284853.s001.zip › SUPPROTING INFORMATION.docx]

**SUPPORTING INFORMATION**

**Mechanistic synergy of hair growth promotion by the *Avicennia marina* extract and its active constituent (avicequinone C) in dermal papilla cells isolated from androgenic alopecia patients**

Woraanong Prugsakit ^1^, Sukanya Numsawat ^2,5^, Ponsawan Netchareonsirisuk ^2,4^, Parkpoom Tengamnuay ^1^, Wanchai De-Eknamkul ^3,4,^*

^1^ Department of Pharmaceutics and Industrial Pharmacy, Faculty of Faculty of Pharmaceutical Sciences, Chulalongkorn University, Bangkok 10330, Thailand; on_wora@hotmail.com; parkpoom.t@chula.ac.th

^2^Department of Biochemistry and Microbiology, Faculty of Pharmaceutical Sciences, Chulalongkorn University, Bangkok 10330, Thailand; ponsawan.n@chula.ac.th; pharmatug@hotmail.com

^3^Department of Pharmacognosy and Pharmaceutical Botany, Faculty of Pharmaceutical Sciences, Chulalongkorn University, Bangkok 10330, Thailand; wanchai.d@chula.ac.th

^4^Natural Product Biotechnology Research Unit, Faculty of Pharmaceutical Sciences, Chulalongkorn University, Bangkok 10330, Thailand; wanchai.d@chula.ac.th; ponsawan.n@chula.ac.th

^5^ Present Address: Bureau of Drug Control, Food and Drug Administration, Ministry of Public Health, Nonthaburi 11000, Thailand

* Corresponding author: Wanchai De-Eknamkul, E-mail: [wanchai.d@chula.ac.th](mailto:wanchai.d@chula.ac.th)

**
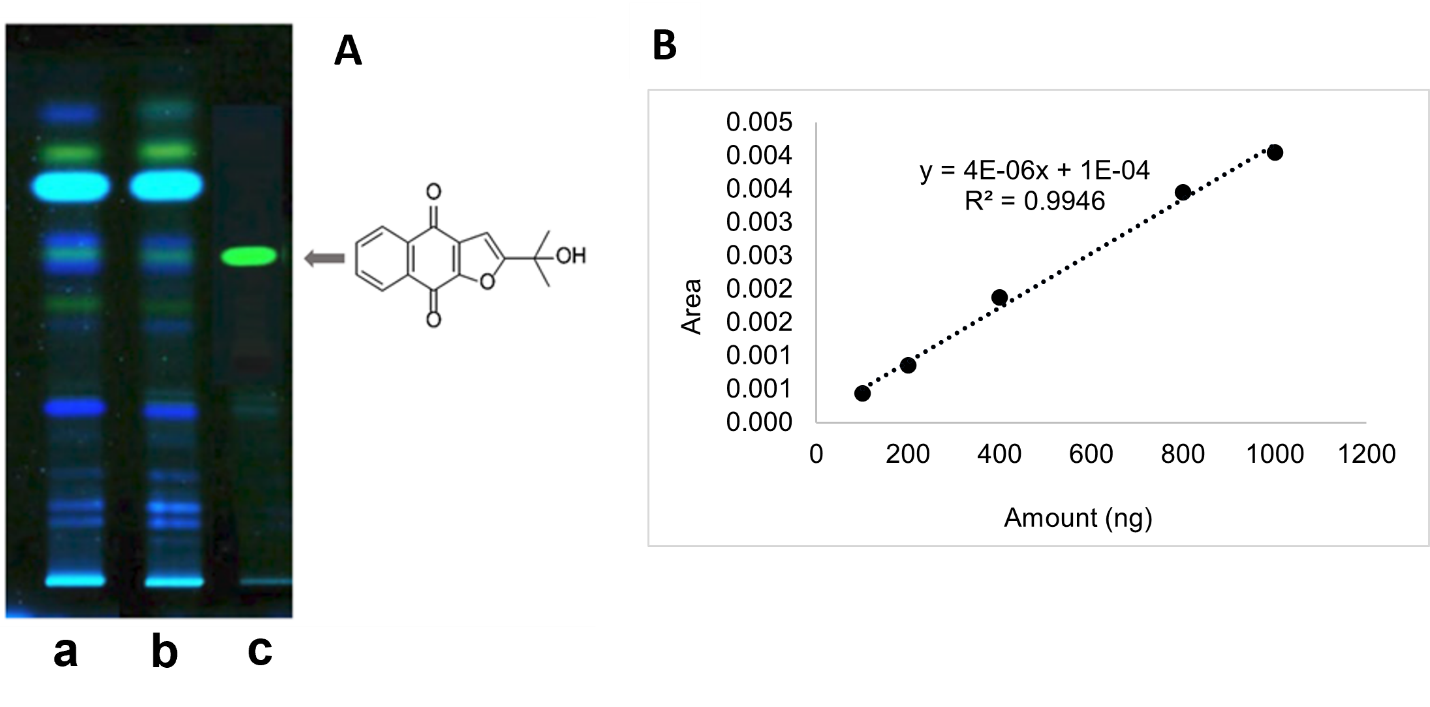
**

**S1A Fig.** TLC-chemical profiles of AM extracts (visualized under UV 366 nm) freshly prepared from the heartwood powder purchased on June 19, 2019 (a), and after storing the extract at -20°C for three years (b), with avicequinone C (AC) used as a marker (c). **S1B Fig.** Calibration of Avicequinone C by TLC densitometric method (scanned at 254 nm) showing linearity range from 100-1000 ng.


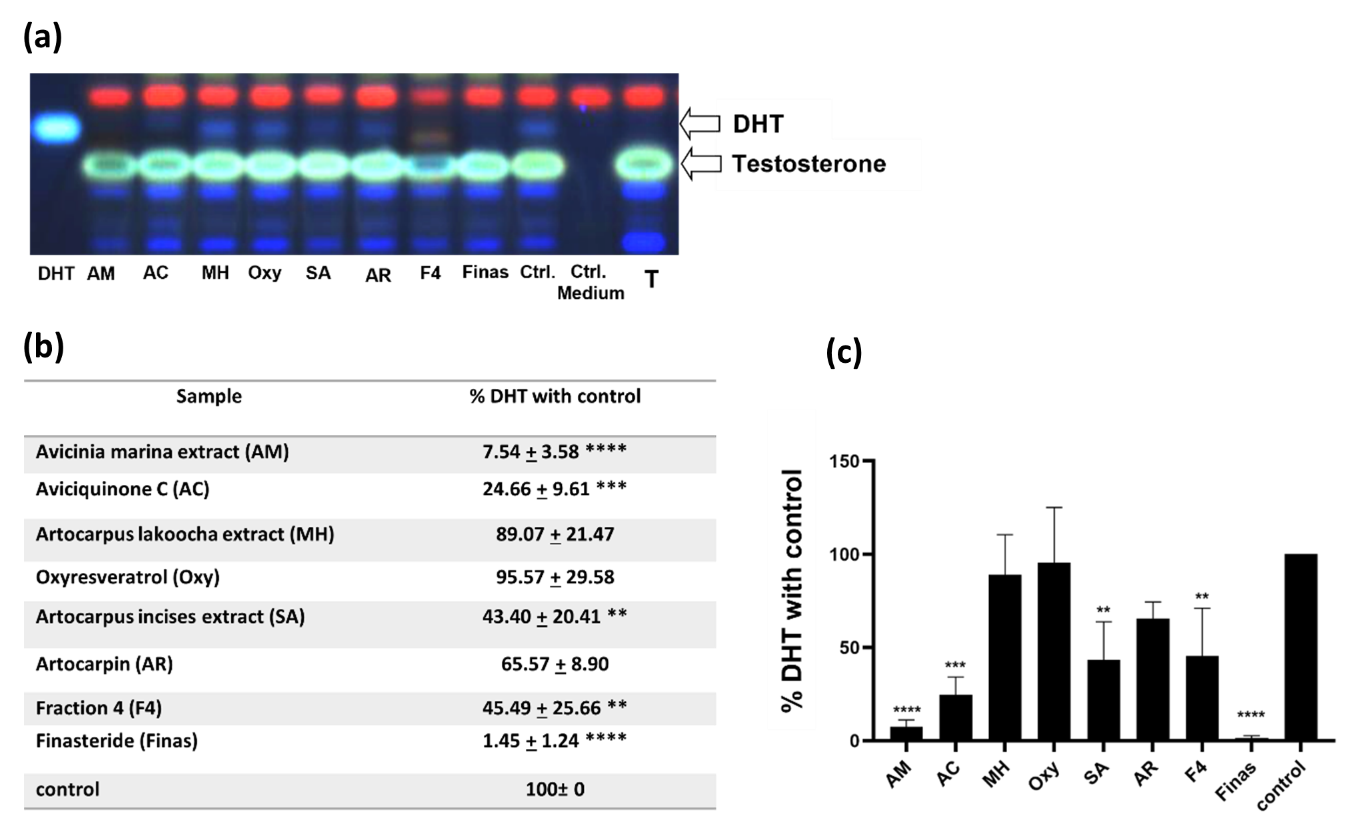


**S2 Fig. DPC cell-based assay determining 5α-R inhibitory activity on TLC technique**. (a) A TLC plate show 5α-R inhibitory activity of AM (10 µg/mL), AC (10 µM), Artocarpus lakoocha extract (MH) (10 µg/mL), oxyresveratrol (Oxy), Artocarpus incises extract (SA) (10 µg/mL), artocarpin (AR) (10 µM), one fraction 4 of AM extract (F4) (10 µM), and finasteride (Finas) (1 µM). The standards dihydrotestosterone (DHT) (3.5 µM) and testosterone (T) (30 µM) are shown as labels. (b) the percentages of DHT formation compared with control cells. (c) The bar graph shows the % of DHT formation in the presence of AM, AC and other samples in relative to the internal control. The data is presented as mean + SD (n=3). ** p < 0.001, *** p < 0.005 and **** p < 0.0001 versus control cells.


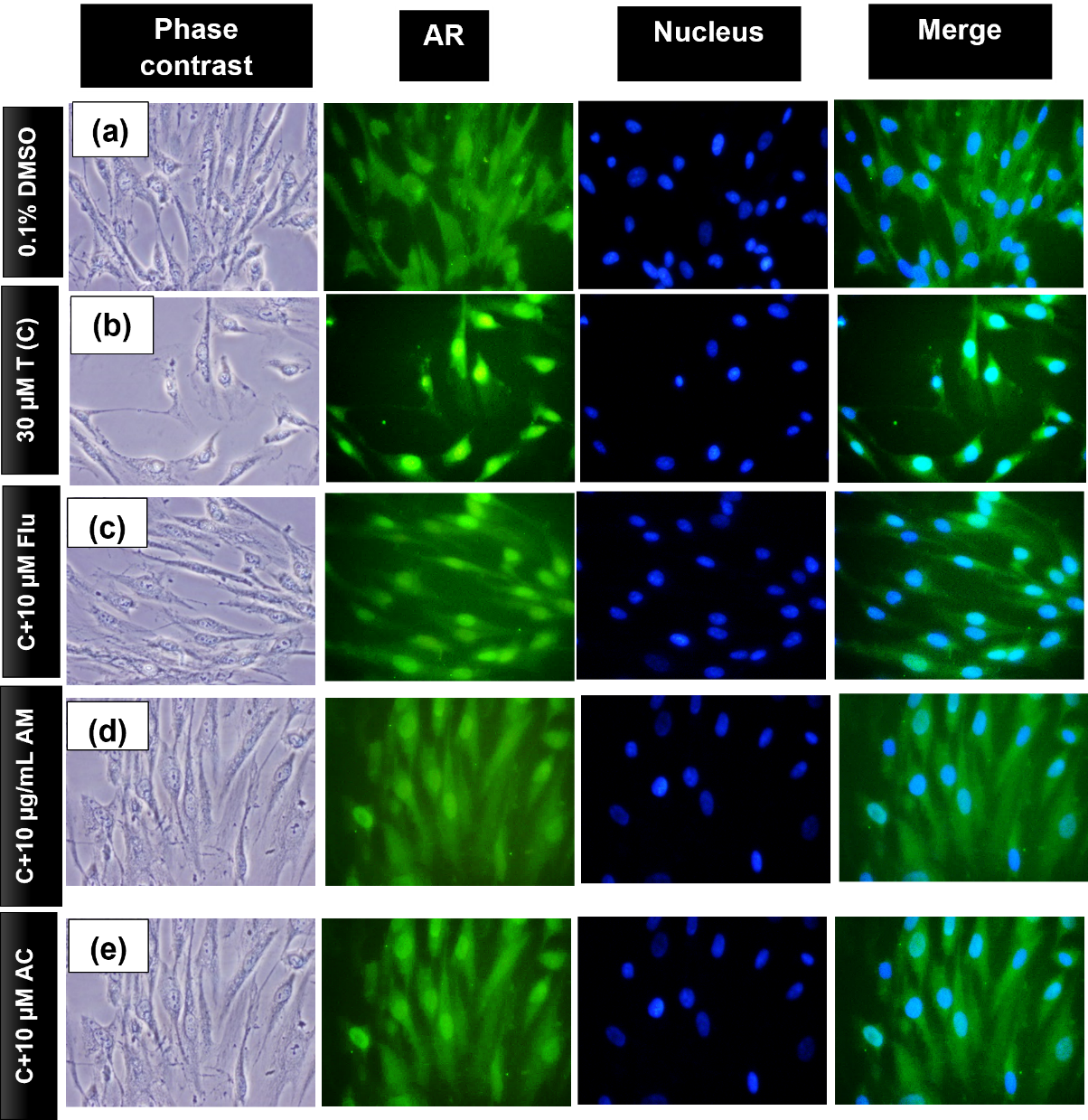


**S3 Fig. The immunofluorescent staining of the androgen receptor (AR) and observed under a Fluorescence-microscope (40x).** For each experimental condition, the first column shows phase contrast of DPCs. The second column shows the AR staining green fluorescence of AR using antibody against AR. The third column shows blue fluorescence nuclear staining by Hoechst dye and the fourth column shows the merged image. a) DPCs were under normal condition (without androgens stimulation), showing the same level intensity of AR staining in both cytoplasm and nucleus. b) DPCs were treated with 30 µM of T for 12 h, AR staining was detected mainly in nucleus. c) DPCs were treated with 10 µM Flutamide and stimulated by 30 µM of T as a positive control. d) DPCs were treated with 10 µg/mL of AM and stimulated by 30 µM of T. e) DPCs were treated with 10 µM AC and stimulated by 30 µM of T.

**
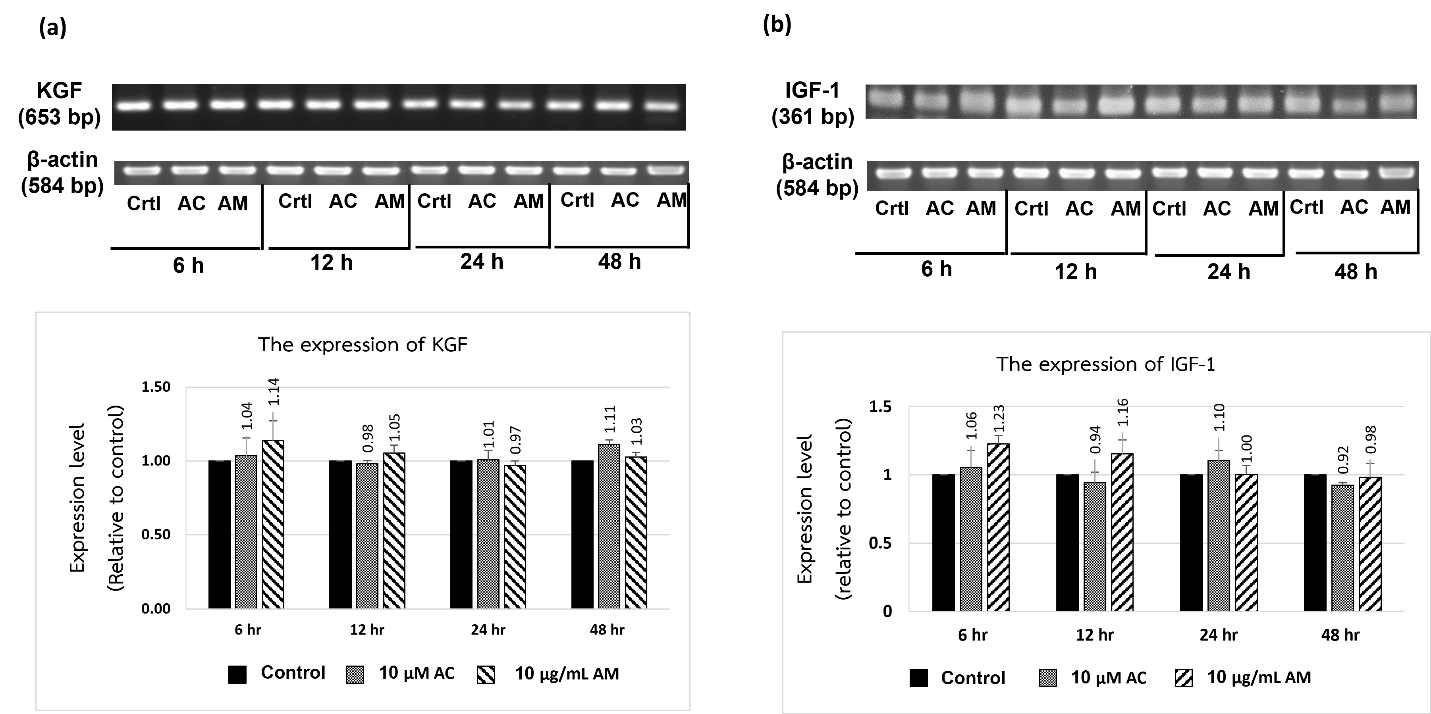
**

**S4 Fig.** **Effect of AM (10 µg/mL) and AC (10 µM) on the expression growth factor gene (KGF and IGF-1).** DPCs were incubated with 30 µM T and treated with AM and AC for various times (6, 12, 24, 48 h) and determined the level of (a) KGF gene expression and (b) IGF-1 gene expression. The expression level of each growth factors was analyzed by using ImageJ 1.53e software. All data were performed in triplicate and presented as mean + SD (n=3).

**
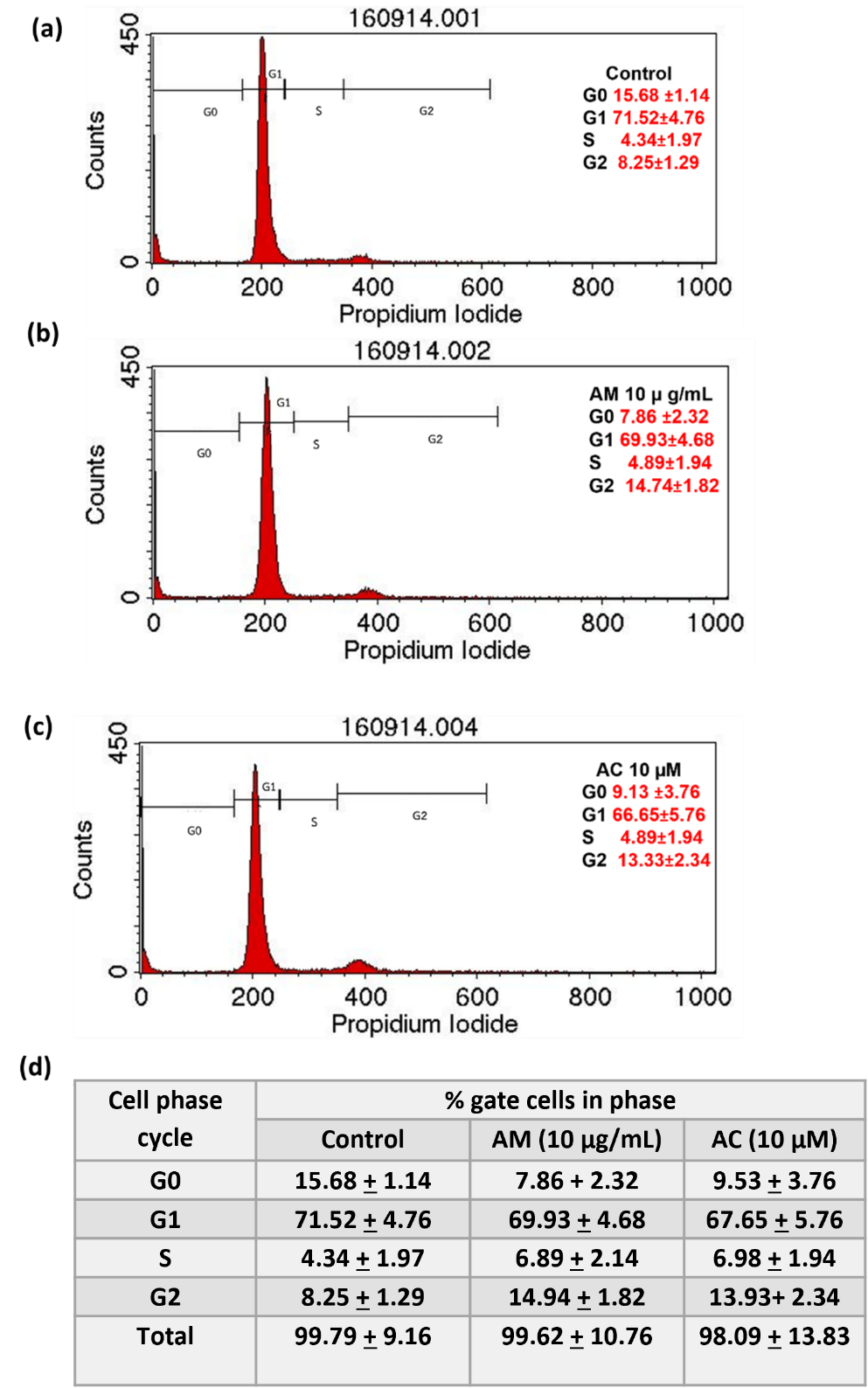
**

**S5 Fig.** **Effect of AM (10 μg/mL) and AC (10 μM) on cell cycle distribution.** The chromatograms show cell cycle distribution of DPCs, (a) control, (b) the cells were treated with AM (10 μg/mL) and (c) the cells were treated with AC (10 μM) for 48h. (c) The data of cell cycle analysis presented % phase of cell cycle. The cells were analyzed for DNA content by a FACSort flow cytometer and evaluated sub G0/G1 phase for DNA distribution in apoptosis process. The data is presented as mean + SD (n=3).

**
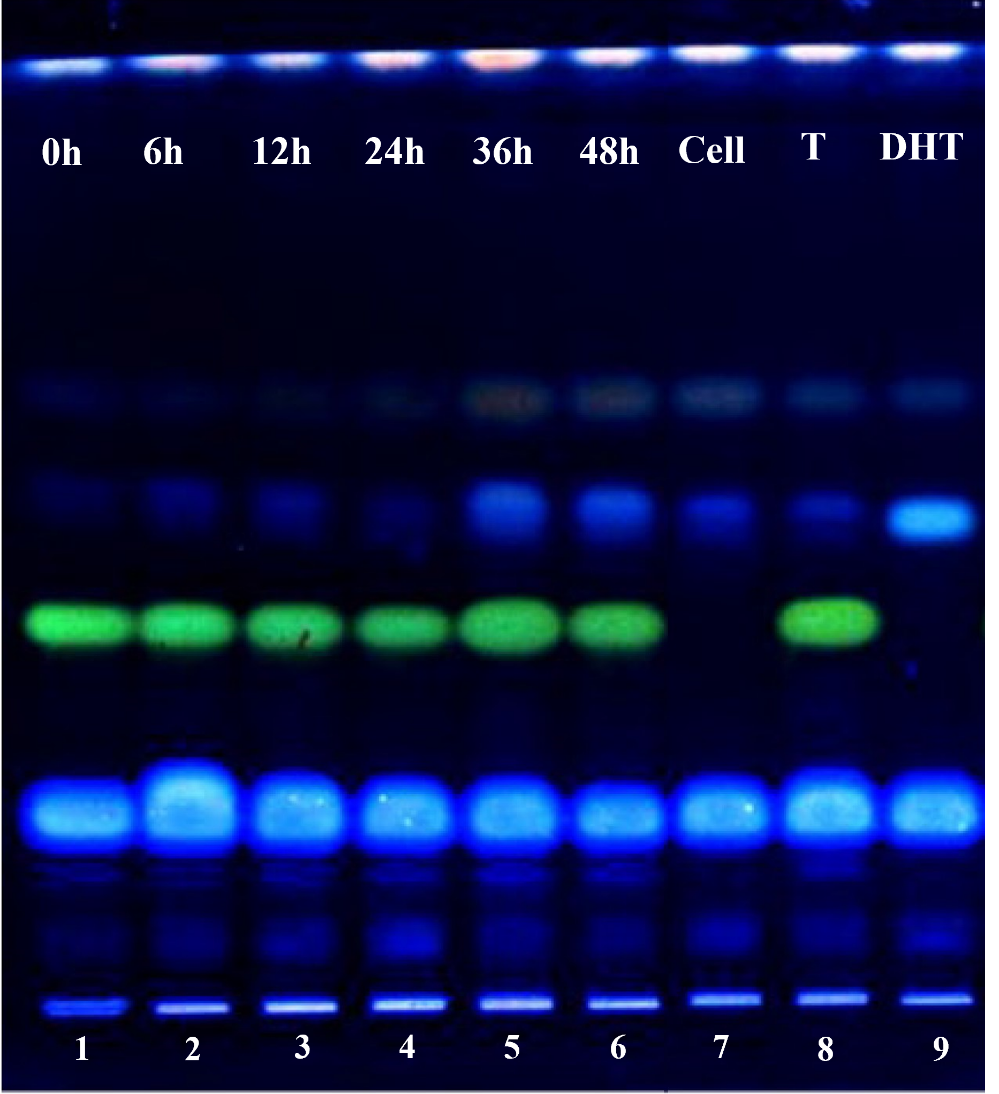
**

**S6 Fig. A TLC plate shows the optimization of 5α-R activity in various time points.** DP primary cells were treated with substrate testosterone to detect the optimum time of dihydrotestosterone formation at 0-48h (lane 1-6). DP primary cells without testosterone (lane 7). Testosterone (lane8) and dihydrotestosterone (lane9).
