# Supplementary material for: Mechanistic synergy of hair growth promotion by the Avicennia marina extract and its active constituent (avicequinone C) in dermal papilla cells isolated from androgenic alopecia patients
Source: PLoS One. 2023 Apr 21;18(4):e0284853. doi: 10.1371/journal.pone.0284853 (PMC10121027; doi:10.1371/journal.pone.0284853)

Fig 7. The effect of the AM extract on the protein expression of the hair growth factors: (HGF, KGF and VEGF)

Fig7a HGF and β-Actin blot

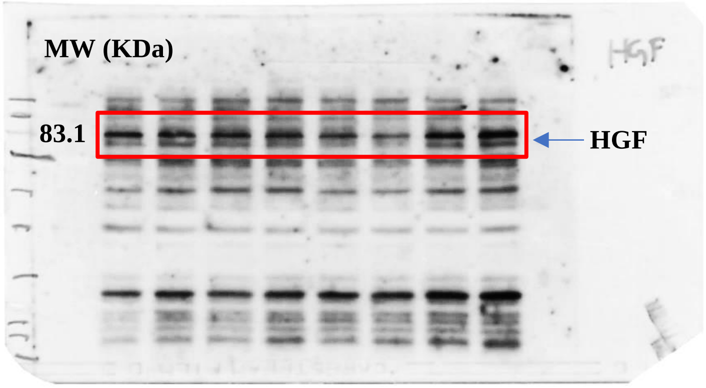

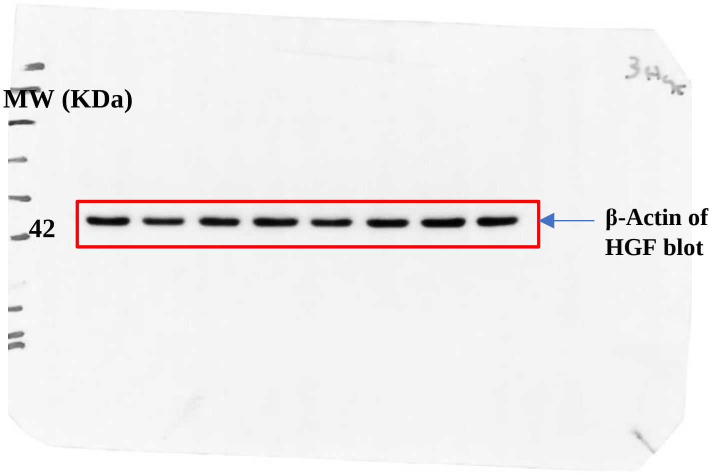

Fig7b KGF and β-Actin blot

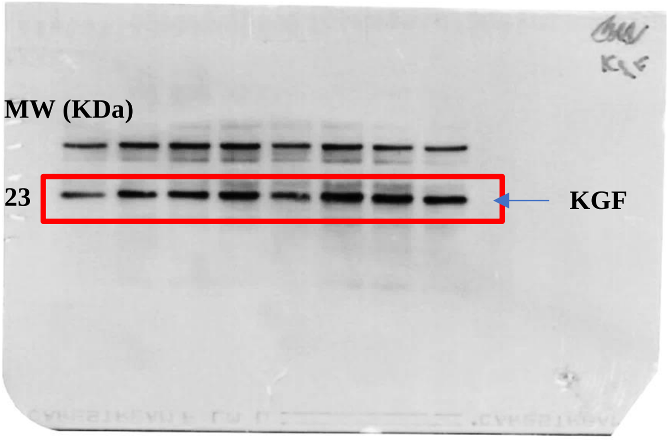

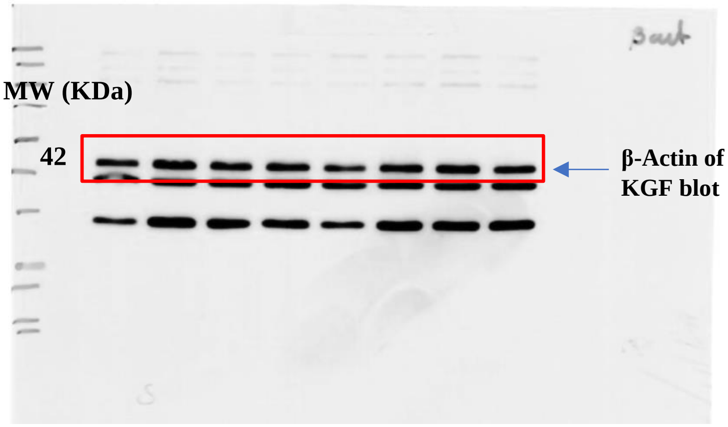

Fig7c VEGF and GAPDH blot

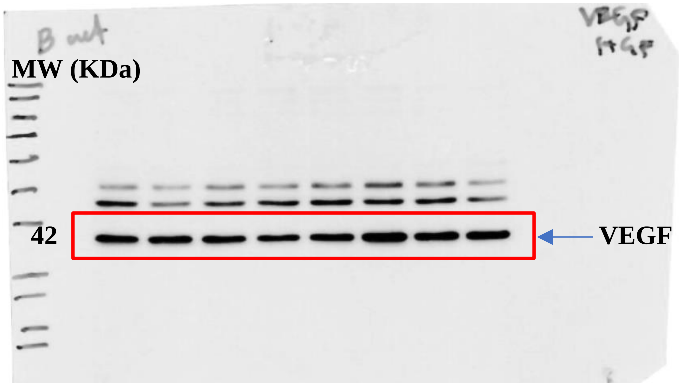

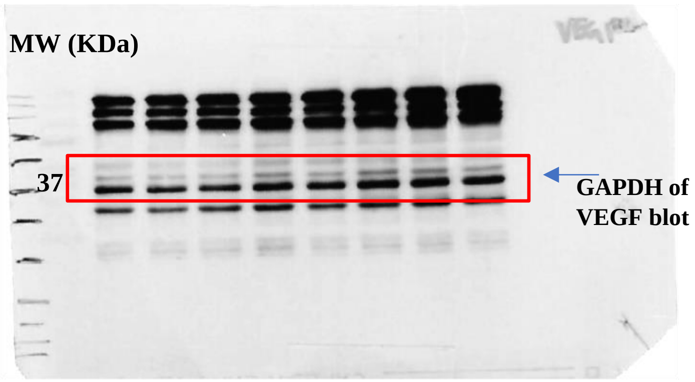

Fig 8. The effect of the AM extract on the protein expression of anti-apoptotic mediators. (Bcl-2, Bax and cleaved caspase-3)

Fig8a Bcl-2 and β-Actin blot

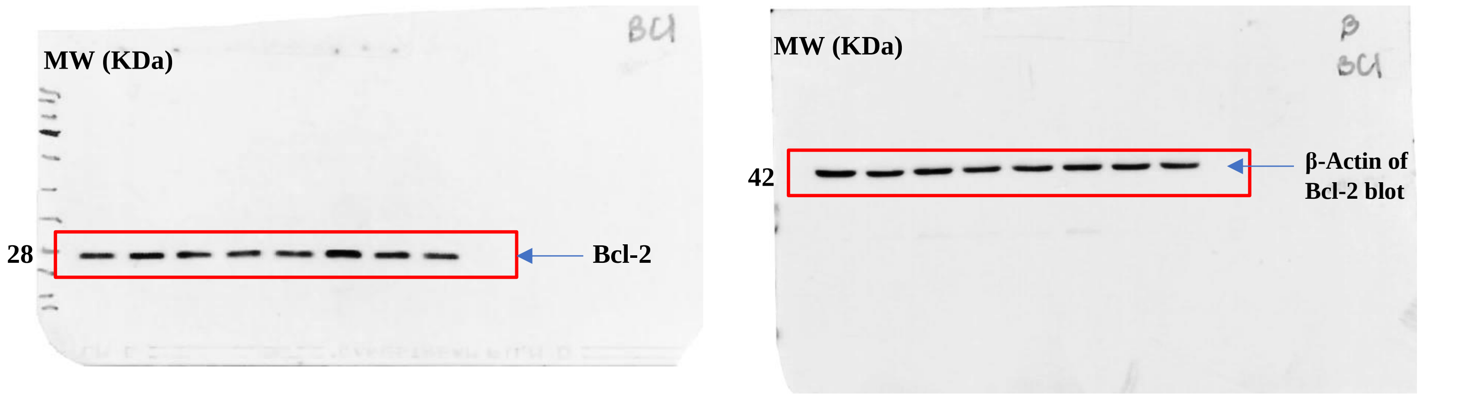

Fig8b Bax and β-Actin blot

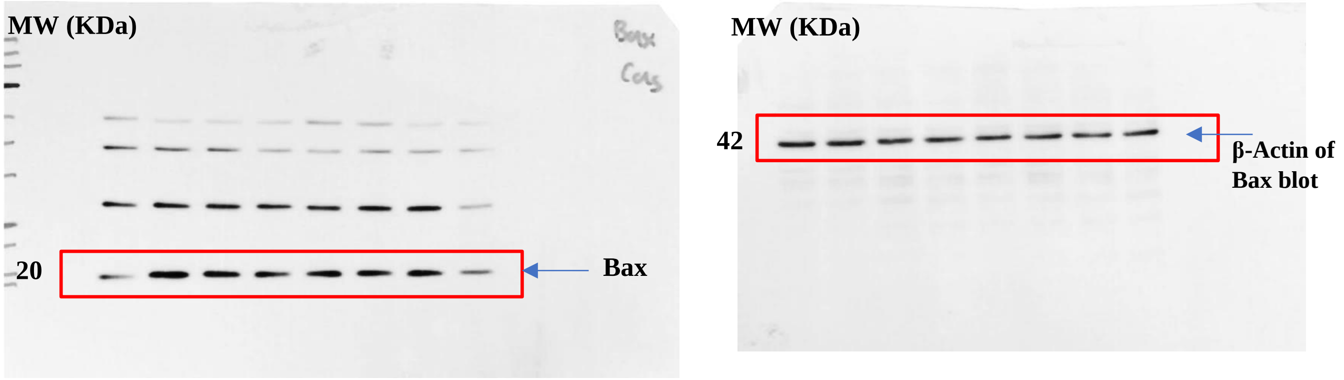

Fig8c cleaved caspase-3 and β-Actin blot

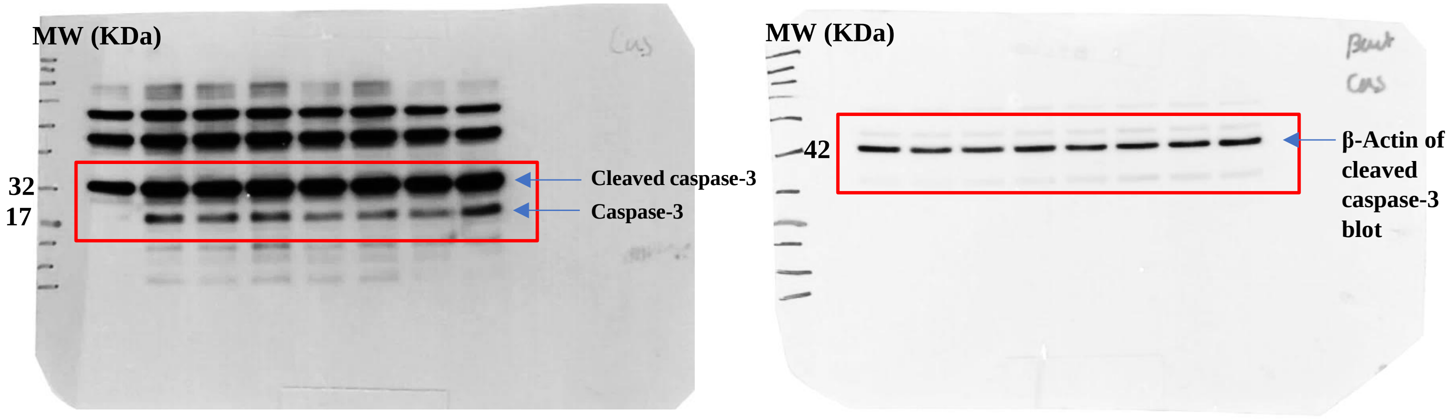

Fig 9. Effect of AM on the protein expression of BMP-4.

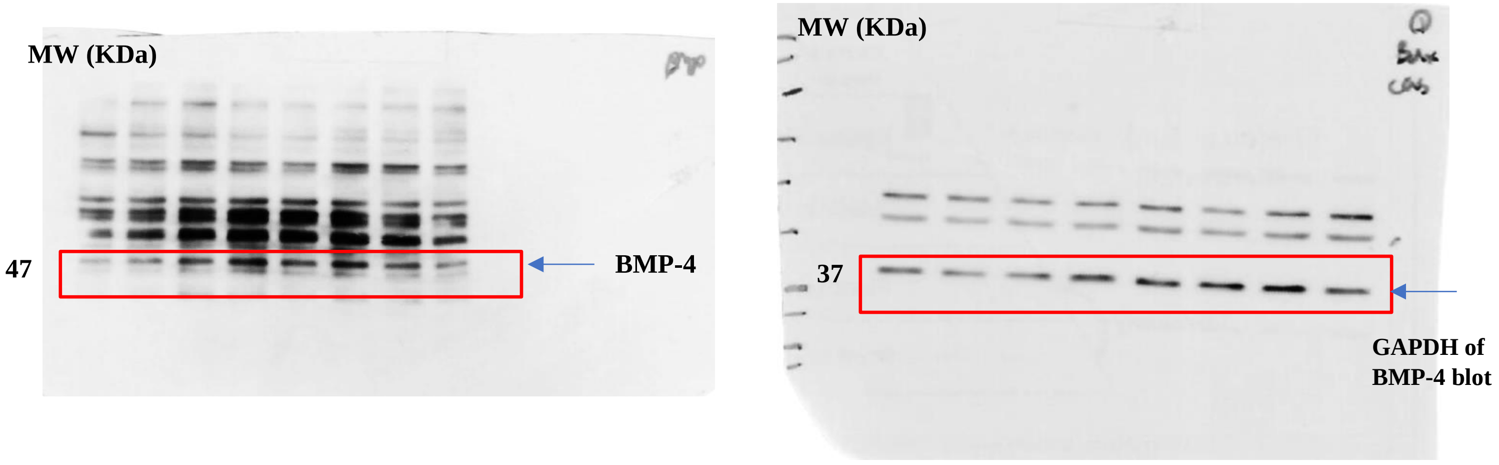

Supplement: S1 Raw images — (PDF) [file pone.0284853.s002.pdf]
