# Supplementary material for: Mechanistic synergy of hair growth promotion by the Avicennia marina extract and its active constituent (avicequinone C) in dermal papilla cells isolated from androgenic alopecia patients
Source: PLoS One. 2023 Apr 21;18(4):e0284853. doi: 10.1371/journal.pone.0284853 (PMC10121027; doi:10.1371/journal.pone.0284853)
Supplement: Attachment — Submitted filename: Reviewer Note.docx [file pone.0284853.s003.docx]

This study is meaningful as a basic study for the development of a hair growth promoting mechanism and androgenic alopecia treatment of *Avicennia marina* extract and its active ingredient (avicequinone C).

We recommended that the following corrections are necessary.

1. There are no results regarding the cytotoxicity of AM and AC. It is necessary to write a separate Fig. In addition, it is necessary to description in detail why the experiment was performed only on low concentrations of the sample with AM (0-40 µg/mL) and AC (0-40 µM), and why the time was measured as 48 hours.
2. When measuring the protein expression of hair growth factor, the AM treatment concentration was effective at 10 µg/mL. When translocation from the cytoplasm to the nucleus was confirmed, the AM treatment concentration was increased by 2 times (20 µg/mL). What is reason?
3. In the cell cycle experiment, are there any results for each AM and AC treatment time period and concentration? What is the rationale for setting AM (10 µg/mL) and AC (10 µM) concentrations? And, what is the rationale for setting 48 hours?
4. Line 438: This may reflect the synergistic effects of the constituents present in the AM extract. → It is necessary to supplement the consideration of other active ingredients present in *Avicennia marina*.
5. Line 240: 366 font size

Line 248: Unit correction required (mM → µM)

Line 478: T?? DHT??

Line 528: in vitro, in vivo in italics

Line 638: 5 font size

Figure 3 (a) 3 Lane, unit correction required mg/mL → µg/mL

Figures 1, 5-8 Numbers and symbols overall poor readability. Needs correction
