# Supplementary material for: Mechanistic synergy of hair growth promotion by the Avicennia marina extract and its active constituent (avicequinone C) in dermal papilla cells isolated from androgenic alopecia patients
Source: PLoS One. 2023 Apr 21;18(4):e0284853. doi: 10.1371/journal.pone.0284853 (PMC10121027; doi:10.1371/journal.pone.0284853)
Supplement: Attachment — Submitted filename: Response to reviewers comments.docx [file pone.0284853.s004.docx]

**Response to Reviewers’ Comments**

**Manuscript:** [PONE-D-22-22814]

**Title:** Mechanistic synergy of hair growth promotion by the Avicennia marina extract and its active constituent (avicequinone C) in dermal papilla cells isolated from androgenic alopecia patients

Reviewers’ Comments

1. There are no results regarding the cytotoxicity of AM and AC. It is necessary to write a separate Fig. In addition, it is necessary to description in detail why the experiment was performed only on low concentrations of the sample with AM (0-40 µg/mL) and AC (0-40 µM), and why the time was measured as 48 hours.

**Response:** We have included the results of cytotoxicity of AM and AC as **Fig. 2** in the main manuscript with describing the detail of the results and adding figure legend. For the question on the measuring time of 48 hours, this was obtained from the time-course study of 5α-R enzyme activity assay (0, 6, 12, 24, 36 and 48 h) using the primary DPCs (**S6 Fig**). The results showed that the activity of 5α-R enzyme was significantly increased during. Therefore, the cytotoxicity of AM and AC treatment was reasonably detected at 48h for cell viability.

**Revision:**

**Line 403-416**: “In order to observe the effects of AM and AC on the cell cycle of the isolated DPCs, the primary cells were first determined for the cytotoxicity of both the extract and active compound by using MTT assay for 48 hr. This incubation time was optimized based on the time-course study of 5α-R enzyme activity assay (0, 6, 12, 24, 36 and 48 h) using the primary DPCs (S6 Fig). The results showed that the treatment of AM at the concentration range of 2.5-20 µg/mL and AC at 2.5-20 µM did not cause significant cytotoxicity on DPCs, compared with the non-treated cells (Fig 2). At the maximum non-toxic concentrations of 20 µg/mL AM and 20 µM AC, the cell viability was observed to be in the range of 80-85% which is lower than the acceptable 90% cell viability. Therefore, we selected the concentrations of 10 µg/mL AM and 10 µM AC for cell cycle analysis and further experiments.”

**Line 428-431:** “**Fig 2**. Cytotoxicity assay of AM and AC on DPCs. DPCs were treated with various concentrations of (**a**) AM (0-40 µg/mL) and (**b**) AC (0-40 µM) for 48 h. Cell viability was evaluated by PrestoBlue® assay. The data is presented as mean + SD (n=3). * p < 0.05 versus non-treated cells.”

2. When measuring the protein expression of hair growth factor, the AM treatment concentration was effective at 10 µg/mL. When translocation from the cytoplasm to the nucleus was confirmed, the AM treatment concentration was increased by 2 times (20 µg/mL). What is the reason?

**Response and Revision:** It was a typo error. We are sorry for the mistake. In fact, the protein expression analysis of hair growth factors and translocation were all treated with AM at 10 µg/mL. We have corrected the concentration from 20 µg/mL to 10 µg/mL in the **Line 324** and **513**.

3. In the cell cycle experiment, are there any results for each AM and AC treatment time period and concentration? What is the rationale for setting AM (10 µg/mL) and AC (10 µM) concentrations? And, what is the rationale for setting 48 hours?

**Response:** For the cell cycle experiment, the concentration and time of AM and AC treatment were decided to set at 10 µg/mL and AC 10 µM which were based on the cytotoxicity result in **Fig2**. AM (10 µg/mL) and AC (10 µM) showed acceptable cell viability more than 90%. For the question on the measuring time of 48 hours, this was obtained from the time-course study of 5α-R enzyme activity assay (0, 6, 12, 24, 36 and 48 h) using the primary DPCs (**S6 Fig**).

**Line 736-737: S6 Fig.** A TLC plate shows the optimization of 5α-R activity in various time points. DP primary cells were treated with substrate testosterone to detect the optimum time of dihydrotestosterone formation at 0-48h (lane 1-6). DP primary cells without testosterone (lane 7). Testosterone (lane8) and dihydrotestosterone (lane9).

**Revision: Line 403-416**: “In order to observe the effects of AM and AC on the cell cycle of the isolated DPCs, the primary cells were first determined for the cytotoxicity of both the extract and active compound by using MTT assay for 48 hr. This incubation time was optimized based on the time-course study of 5α-R enzyme activity assay (0, 6, 12, 24, 36 and 48 h) using the primary DPCs (S6 Fig). The results showed that the treatment of AM at the concentration range of 2.5-20 µg/mL and AC at 2.5-20 µM did not cause significant cytotoxicity on DPCs, compared with the non-treated cells (Fig 2). At the maximum non-toxic concentrations of 20 µg/mL AM and 20 µM AC, the cell viability was observed to be in the range of 80-85% which is lower than the acceptable 90% cell viability. Therefore, we selected the concentrations of 10 µg/mL AM and 10 µM AC for cell cycle analysis and further experiments.”

4. Line 438: This may reflect the synergistic effects of the constituents present in the AM extract. → It is necessary to supplement the consideration of other active ingredients present in *Avicennia marina*.

**Response** This sentence intended to explain the observed results obtained from the AM extract. However, we agree that an additional sentence should be added to make it clearer.

**Revision: Line 453-456:** “This may reflect the synergistic effects of the constituents present in the AM extract [40]. To prove this, however, it is necessary to find other active constituents in AM, followed by observing the 5α-R inhibitory activity of their combinations with AC.”

5. Line 240: 366 font size

**Revision:** We have revised 366 font size.

Line 248: Unit correction required (mM → µM)

**Revision:** We have corrected the unit from mM to µM

Line 478: T?? DHT??

**Revision:** We have revised line 496-497 “These results suggest that the AM extract and the AC were able to inhibit both DHT-AR and T-AR complex formation.

Line 528: in vitro, in vivo in italics

**Revision:** We have revised italics font in line 545

Line 638: 5 font size

**Revision:** We have revised in line 655.

Figure 3 (a) 3 Lane, unit correction required mg/mL → µg/mL

**Revision:** The unit of mg/mL has been changed to µg/mL in Fig. 4

Figures 1, 5-8 Numbers and symbols overall poor readability. Needs correction

**Revision:** The numbers and symbols were all corrected.
